# Identification, Characterization, and Expression Analysis of a FMRFamide-Like Peptide Gene in the Common Chinese Cuttlefish (*Sepiella japonica*)

**DOI:** 10.3390/molecules23040742

**Published:** 2018-03-23

**Authors:** Ying Li, Zihao Cao, Haifeng Li, Huihui Liu, Zhenming Lü, Changfeng Chi

**Affiliations:** National and Provincial Joint Laboratory of Exploration and Utilization of Marine Aquatic Genetic Resources, National Engineering Research Center of Marine Facilities Aquaculture, School of Marine Science and Technology, Zhejiang Ocean University, 1st Haidanan Road, Changzhi Island, Lincheng, Zhoushan 316022, China; y.lisalee@foxmail.com (Y.L.); expandable1@126.com (Z.C.); chicf@zjou.edu.cn (Ha.L.); liuhuihui2004@126.com (Hu.L.); nblzmnb@163.com (Z.L.)

**Keywords:** neuropeptide, FMRFamide, mollusk, cuttlefish, *Sepiella japonica*

## Abstract

The peptide FMRFamide is one of the well-known peptides involved in multiple physiological processes in the phylum Mollusca. In this study, a FMRFamide gene (GenBank accession No. KJ933411) was identified in a cuttlefish species called *Sepiella japonica* and was designated as SjFMRFamide. The total length of the SjFMRFamide sequence was found to be 1880 bp while the open reading frame contained 996 bp encoding a protein of 331 amino acid residues with a predicted isoelectric point (*pI*) and molecular weight (MW) of 9.18 and 38.8 kDa along with a 333 bp 5′-untranslated region (UTR) and 551 bp 3′-UTR. The deduced SjFMRFamide precursor protein contains one signal peptide and expresses four kinds FMRFamide-related peptides including a single copy of FLRFamide, ALSGDAFLRFamide, and FIRFamide and multiple copies of FMRFamide. Results of phylogenetic relation analysis strongly indicated that the sequence of this gene shares high identity with the genes of known FMRFamides. Spatial expression analysis indicated the highest mRNA expression of SjFMRFamide in the brain of male and female cuttlefishes among the eight tissues analyzed. An in situ hybridization assay of the brain indicated that SjFMRFamide was transcribed in several functional lobes, which suggests that it might be related to many physiological regulatory mechanisms. This is the first study describing FMRFamide in *S. japonica* and the results may contribute to future studies of neuropeptide evolution or may prove useful for the development of aquaculture methods for this cuttlefish species.

## 1. Introduction

Since FMRFamide [1] was discovered and named the molluscan cardio acceleratory peptide in 1977, all kinds of neuropeptides that share the C-terminal RFamide have been identified in animals ranging from cnidarians to vertebrates [2]. This peptide along with its structurally-related neuroactive peptides, FMRFamide-like peptides (FLPs), represents a widely investigated family of neuropeptides in both invertebrate and vertebrate phyla such as Nematoda, Annelida, Arthropoda, Chordata, insects, and mollusks [3,4,5]. These neuropeptides serve as important neurotransmitters or neuromodulators in the nervous system and are involved in many physiological functions [6,7].

With the refinement of methodologies for peptide separation, purification, and sequencing, various kinds of new relevant peptides have been discovered in mollusks. Five unique genes including the FMRFamide gene, cholecystokinin/sulfakinin (CCK/SK)-related gene, LFRFamide gene, luqin gene, and the neuropeptide F (NPF) gene, which code for RFamide peptides, have been identified [8]. Many studies that have focused on FLP distribution have indicated that these peptides are widely expressed in the central and peripheral nervous systems (CNS and PNS). In gastropods, FMRFamide immunoreactivity has been confirmed in the nerves and ganglia of *Helix aspersa* [9]. In Bivalvia, FMRFamide immunoreactivity was demonstrated in intraganglionic regions of *Mytilus eduli* [10] and ganglia of *Placopecten magellanicus* [11]. In cephalopods, FMRFamide immunostaining has been demonstrated in the CNS of *Sepia officinalis* [12] and *Octopus vulgaris* [13]. Physiological studies on FMRFamides revealed that these peptides are involved in chromatophore regulation [14,15], reproduction [6,16], and control of the neuroendocrine optic gland [16]. 

*Sepiella japonica* is an economically important cephalopod in the East China Sea and plays a key role in the marine ecosystem. This species has been one the most famous seafood of the East China Sea, but the resources began to decline in the 1970s because of overfishing and environmental changes. Because of this situation, our research group began to work on the resource restoration from 2003 to 2008 and now we can culture the cuttlefish artificially. During the last several years, we found that the sexual maturity of artificially cultured cuttlefish starts earlier than that of the wild ones [17]. This phenomenon that the cultured cuttlefish is susceptible to precocious puberty was also reported by Zheng et al. in 2010 [18]. The peptide under consideration here is known to be involved in reproduction and thus is closely related to sexual maturity of these cephalopods. In our present study, the full-length cDNA of FMRFamide (designated as SjFMRFamide) from *S. japonica* was cloned followed by phylogenetic analyses and investigation of the expression pattern of SjFMRFamide mRNA in tissues. These results will clarify the function and possible role of FMRFamide in the development of *S. japonica* individuals. 

## 2. Results and Discussion

### 2.1. Whole-Length Sequence Analysis and Protein Structural Prediction

**Figure 1:**
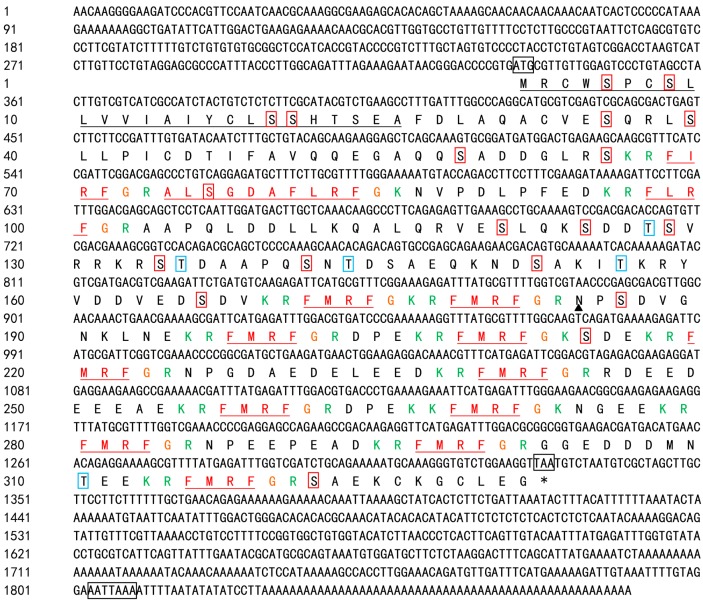
Full-length cDNA sequence and deduced amino acid sequence of SjFMRFamide from *S. japonica*. The putative signal peptide is underlined in black. The predicted FaRPs are marked in red and underlined. The basic or dibasic cleavage sites are green and the glycines used for C-terminal amidation are highlighted in orange. The putative start codon, stop codons, and the polyadenylation signal (AATAAA) are each enclosed in a box. An *N*-glycosylated site is indicated with a black triangle and phosphorylation sites are represented by solid lines in which serines are marked in red and threonines are highlighted in blue.

**Figure 2:**
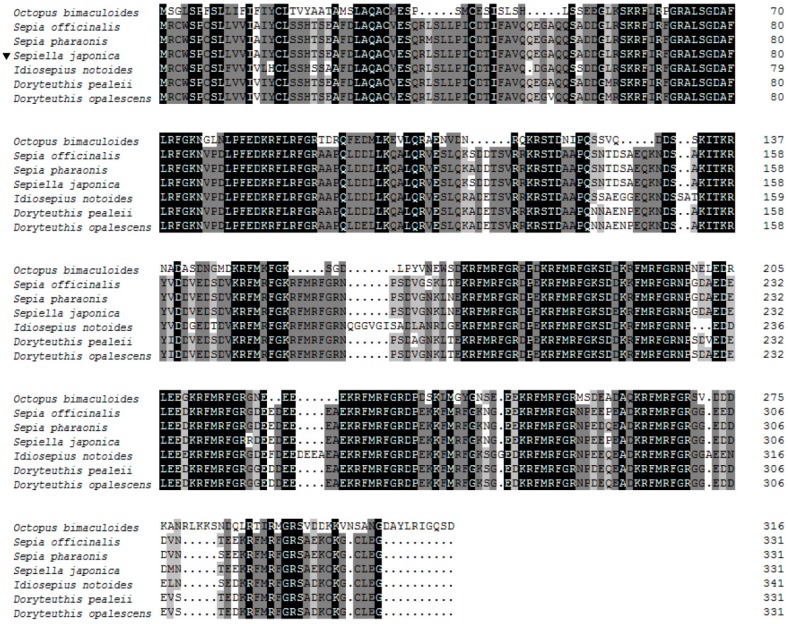
Multiple alignment of the deduced amino acid sequences of FMRFamides. Residues identical in all seven sequences are boxed in black. Conserved substitutions are boxed in gray. GenBank accession numbers of the sequences include: *Octopus bimaculoides* (XP_014775928.1), *Sepia officinalis* (CAA72116.1), *Sepia pharaonis* (AQM50872.1), *Sepiella japonica* (AJT49288.1), *Idiosepius notoides* (ACP39631.1), *Doryteuthis pealeii* (ACI22791.1), and *Doryteuthis opalescens* (AAG22544.1).

Assembly of degenerate and rapid amplification of cDNA ends (RACE) PCR fragments yielded an mRNA sequence 1880 bp long (GenBank accession No. KJ933411) with an open reading frame (ORF) of 996 bp encoding 331 amino acid residues (see Figure 1). There was a 333 bp untranslated region (UTR) at the 5′ end as well as a 551 bp UTR at the 3′ end. The predicted protein molecular weight (MW) was 38.8 kDa and a predicted isoelectric point (*pI*) was 9.18. The BLASTn analysis indicated that the coding sequence shares high similarity and identity with known FMRFamides (see Figure 2).

FMRFamide-related peptides (FaRPs) were first found in bivalves and characterized by RFamide at the C-terminus of the polypeptide [1]. FaRPs belong to a subfamily of RFamide-related peptides [19,20]. FaRPs are expressed by a single gene in Cephalopoda [21]. Martin and Chin isolated the polypeptide members of this subfamily including FMRFamide, FLRFamide, FIRFamide, and ALSGDAFLRFamide from *Octopus vulgaris* and *Loligo opalescens* [22,23]. The SignalP software, v.4.0, showed that there was a likely signal peptide of 25 aa and four different FaRPs: a single copy of FLRFamide, the decapeptide ALSGDAFLRFamide, FIRFamide, and multiple copies of FMRFamide. These peptides have been identified in several species [1,21,24]. The C-terminus of each peptide ends with Arg-Phe, followed by a Gly, which indicated post-translational amidation of phenylalanine [25]. Lys-Arg, Lys-Lys, Arg, or Lys at the flank of the FaRPs may serve as internal proteolytic cleavage sites in post-translational processing. The protein contains several potential sites for post-translational modifications: an N-linked glycosylation sites (aa position 184) and 24 phosphorylation sites including 19 serine sites and five threonine sites (see Figure 1). Transmembrane structure analysis indicates that the protein is an extra membranous protein since it presumably acts outside the cell membrane.

### 2.2. A Blast Search and Phylogenetic-Tree Construction

Online BLAST (NCBI) was used to investigate the similarity of the predicted SjFMRFamide protein to those of other organisms. Related sequences were downloaded and used in an alignment performed using ClustalW [26]. The analysis in ClustalW revealed that the deduced amino acid sequence of SjFMRFamide showed high similarity with other known FMRFamides (see Figure 2). For example, it shares 98% identity with FMRFamide from *S. officinalis* (331 aa, CAA72116.1), 98% identity with *Sepia pharaonis* (331 aa, AQM50872.1), 92% identity with *Doryteuthis opalescens* (331 aa, AAG22544.1), 92% identity with *Doryteuthis pealeii* (331 aa, ACI22791.1), 83% identity with *Idiosepius notoides* (341 aa, ACP39631.1), and 54% identity with *Octopus bimaculoides* (317 aa, XP_014775928.1). This finding showed that FMRFamide in *S. japonica* is very similar to FMRFamide in *S. officinalis* and *S. pharaonis.* We predicted that the RF residues at the C-terminus of the FMRFamide peptides are evolutionarily conserved and a variety of N-terminal residues might be related to multiple physiological roles in animals [7]. 

**Figure 3:**
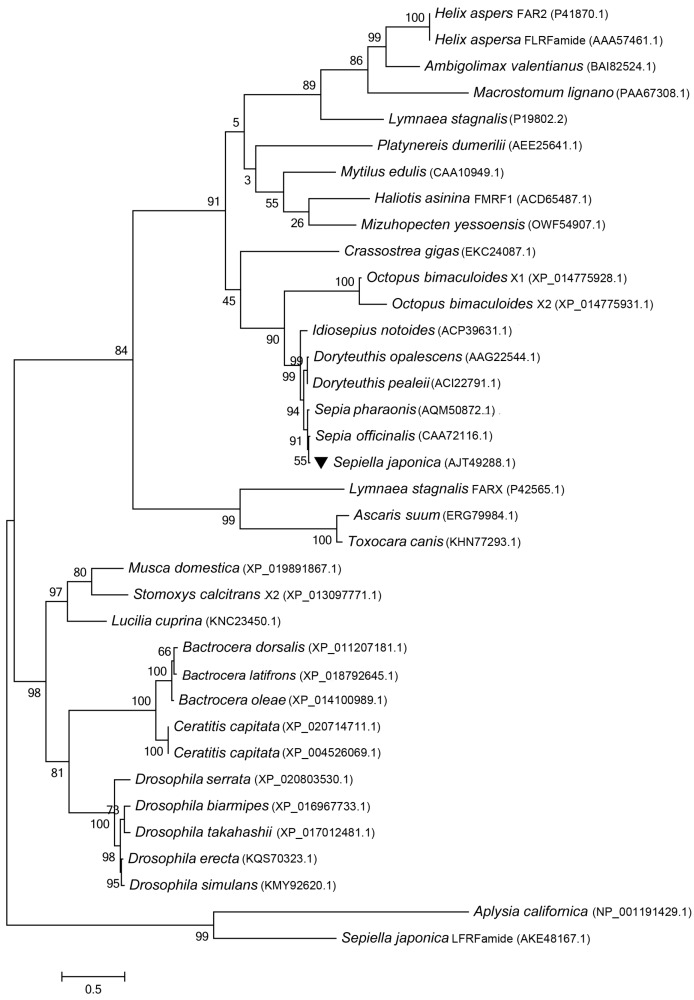
A phylogenetic tree constructed with SjFMRFamide and 36 kinds of RFamide sequences by using the Maximum Likelihood method (MEGA 6.0). All protein sequences were obtained from GenBank and the accession numbers are presented in parentheses. The topological stability of the tree was achieved by running 1000 bootstrapping replications. Bootstrap values (%) are indicated by numbers at the nodes. The scale for branch length is shown below the tree.

A second transcript (mRNA) coding for FaRPs has been found to exist in some mollusks. In *Lymnaea stagnalis*, mRNA 1 included exons E1 and E2 and mRNA 2 included exons E1, E3, E4, and E5 [27]. In *H. aspersa*, two mRNAs have also been characterized [28]. The phylogenetic tree (see Figure 3) showed that SjFMRFamide grouped with almost all the mollusks and clustered together as a subgroup. *S. japonica* has a tight evolutionary relation with *S. officinalis* and *S. pharaonis*. The second transcripts of FMRFamides from arthropods acted as an outgroup. Until now, the second mRNA of the FMRFamide gene has not been identified in cephalopods.

### 2.3. The Expression Pattern of SjFMRFamide mRNA in Different Tissues.

**Figure 4:**
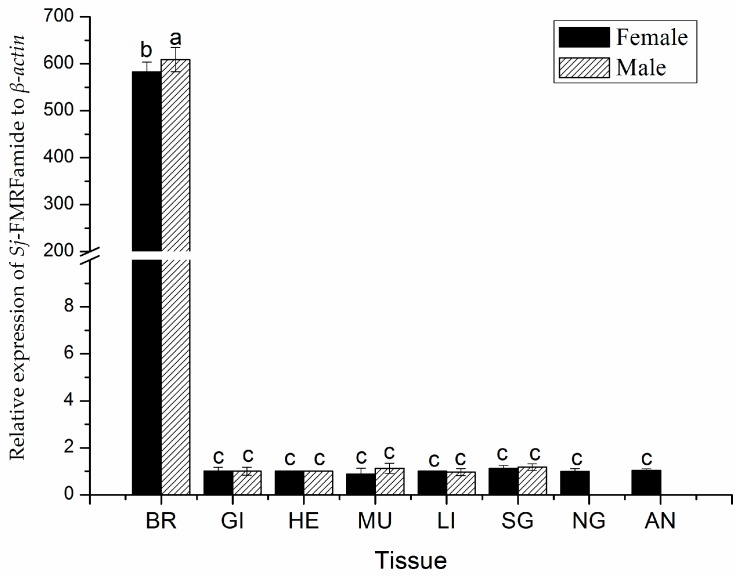
Distribution of SjFMRFamide mRNA in different *S. japonica* tissues.The analyses were conducted in eight different tissues including the brain (BR), gill (GI), heart (HE), muscle (MU), liver (LI), sexual gland (SG, including testis & ovary), nidamental gland (NG), and accessory nidamental gland (AN). β-Actin served as a reference gene. Different letters on the bars represent statistically significant differences among the tissues in the LSD multiple comparison test of SPSS [31] (*p* < 0.05).

Some studies have shown that neuropeptides like gonadotropin-releasing hormone (GnRH) may be expressed in the ovary of *S. officinalis* [29]. Nonetheless, in the squid (*L. edulis*), RT-PCR and Southern blot analysis have shown no transcript except in the CNS [30]. In cephalopods, other studies on FMRFamide have mainly focused on the distribution of FaRPs. In our study, the expression of SjFMRFamide in various tissues of male and female cuttlefishes was detected by using quantitative RT-PCR (qRT-PCR). The expression of SjFMRFamide was detected in all eight tissues analyzed including the brain, gill, heart, muscle, liver, sexual gland (including testis & ovary), nidamental gland, and accessory nidamental gland. The heart served as a relative standard, which is shown in Figure 4. We used qRT-PCR analysis with β-Actin as an internal control. SjFMRFamide showed widely different levels of expression in the eight tissues while the highest expression was observed in the brain with approximately 609 times in males and 582 times in females. The mRNA expression of SjFMRFamide in the brain was significantly higher in the male cuttlefishes than in female cuttlefishes. In addition, the expression was significantly higher in the brain than in other tissues (*p* < 0.05).

### 2.4. In Situ Hybridization of the SjFMRFamide mRNA for Expression Analysis 

**Figure 5:**
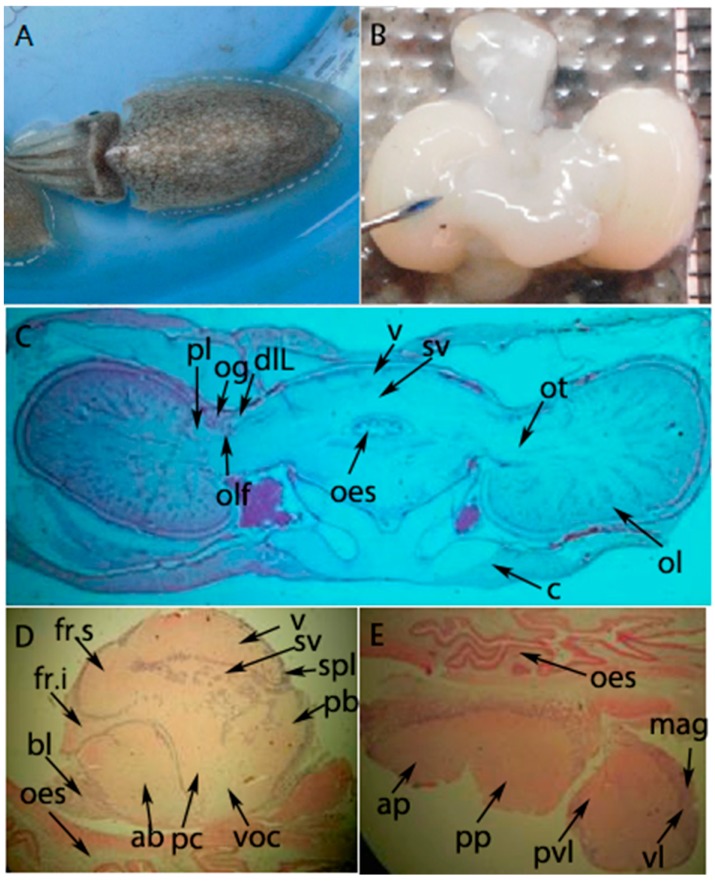
Brain structure of *S. japonica* [33]. (**A**) A whole figure of live *S. japonica*, (**B**) fresh brain after dissection (the needle points to the location of optic gland in the brain), (**C**) transection of the brain (the above part is the cuttlefish back), (**D**) supraesophageal mass (longitudinal section, left side is anterior), (**E**) subesophageal mass (longitudinal section, left side is anterior). Abbreviations: pl, peduncle lobe; og, optic gland; dlL, dorsal lateral lobe; olf, olfactory lobe; v, vertical lobe; sv, subvertical lobe; oes, esophagus; ot, optic tract; ol, optic lobe; c, cartilage; fr.s, superior frontal lobe; fr.i, inferior frontal lobe; bl, brachial lobe; ab, anterior basal lobe; pc, precommissural lobe; voc, ventral optic commissure; spl, subpeduncle lobe; pb, posterior basal lobe; ap, anterior pedal lobe; pp, posterior pedal lobe; pvl, palliorisceral lobe; mag, magnocellular lobe.

**Figure 6:**
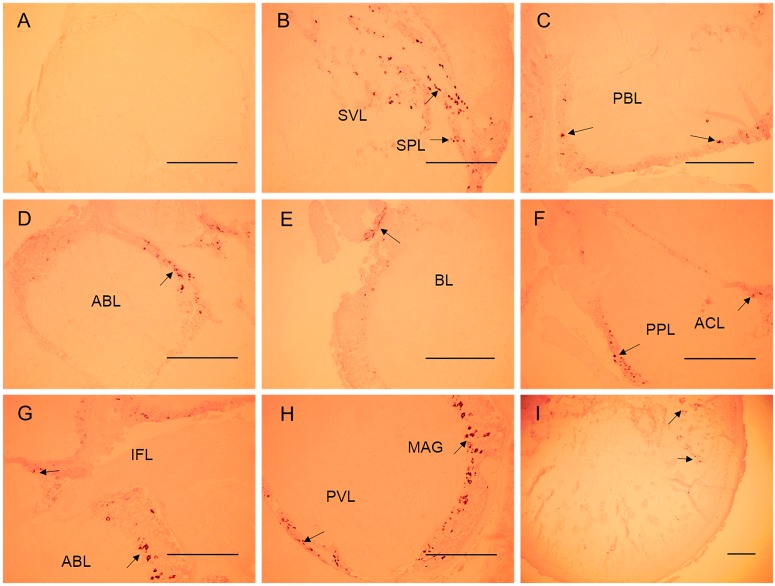
Localization of SjFMRFamide mRNA in the brain of *S. japonica*. (**A**) A medial sagittal section of the anterior basal lobe stained with the sense SjFMRFamide probe, (**B**) the sub-vertical lobe, (**C**) posterior basal lobe, (**D**) anterior basal lobe, (**E**) brachial lobe, (**F**) posterior basal lobe, (**G**) inferior frontal lobe, (**H**) parabrachial lobe, and (**I**) optic lobe. Abbreviations: ABL, anterior basal lobe; ACL, anterior chromatophore lobe; BL, brachial lobe; IFL, inferior frontal lobe; MAG, magnocellular lobe; PBL, posterior basal lobe; PVL, palliorisceral lobe; SFL, superior frontal lobe; SPL, subpeduncle lobe; SVL, subvertical lobe. The black arrows indicate positive signals. Scale bars: 500 μm.

The specific expression of neuropeptides in the cephalopod CNS may be related to their physiological functions [32]. On the basis of qRT-PCR results, the spatial expression profile of SjFMRFamide mRNA in *S. japonica* was investigated next. According to previous research [17,33], we know that the brain of *S. japonica* consists of three parts including the supraesophageal mass, subesophageal mass, and optic lobes (see Figure 5). The major results on SjFMRFamide mRNA expression in the cuttlefish brain consist of three parts (see Figure 6). First, various intensity levels of staining were observed and the supraesophageal mass, sub-peduncle lobe, and sub-vertical lobe showed the strongest staining. The anterior and posterior basal lobes were second in this regard and the inferior frontal lobe manifested the weakest staining. Second, this staining was detected in the subesophageal mass, brachial lobe, pedal lobe, magnocellular lobe, and palliorisceral lobe. Third, optic lobes also showed positive labeling via the SjFMRFamide probe in the medulla. These results are similar to those from immunohistochemical analysis of FaRPs and in situ hybridization performed in adult *S. officinalis* [13,34] and other cephalopods [35,36]. In cephalopods, FMRFamide plays a role in reproduction [13,16]. Therefore, we propose that SjFMRFamide may have a similar physiological function in the reproduction of *S. japonica*.

## 3. Experimental Section

### 3.1. Animals

Mature cuttlefishes (*S. japonica*, 80–180 g) were sampled at a local industrial breeding nursery (Dongji Island, Zhoushan, China) and were immediately shipped to the University. The cuttlefishes were raised with salinity of 28–30% at 23–25 °C with a photoperiod of 14 h light versus 10 h dark every day. The cuttlefishes were fed with prawns twice per day. Adult matured cuttlefishes were used for preparation of brain sections for the in situ hybridization experiment. All the in vivo tests were carried out at the School of Marine Science and Technology of Zhejiang Ocean University (China), which obtained the permission for performing the research protocols and all animal experiments conducted during the present study from the ethics committee of Zhejiang Ocean University. All experimental procedures were conducted under the oversight and approval of the Academy of Experimental Animal Center of Zhejiang Ocean University and in strict accordance with the NIH Guide for the Care and Use of Laboratory Animals (NIH, 2002).

### 3.2. RNA Extraction and cDNA Full-Length Amplification

The cuttlefishes were anesthetized with 0.1% ethanol for 5 min before quick dissection. Various tissues of the brain, gills, heart, muscle, liver, testis, ovary, nidamental gland, and accessory nidamental gland were dissected, snap-frozen in liquid nitrogen, and then stored at −80 °C until RNA preparation. TRIzol lysis buffer (Takara Bio Inc., Otsu, Kyoto, Japan) was employed to obtain total RNA from different tissues. Afterward, 100 mg of extracted tissue in 1 mL of TRIzol was thoroughly ground up with an electric homogenizer and kept for 5 min at room temperature. Then, 200 μL of chloroform was added. This was followed by violent shaking for 15 s and incubation for 5 min. The supernatant was collected after centrifugation at 12,000× *g* at 4 °C for 15 min, mixed with an equal volume of isopropanol, and kept at −20 °C overnight. The following day, the solution was centrifuged at 12,000× *g* at 4 °C for 10 min and the supernatant was discarded. The resulting precipitate was washed twice with 1 mL of fresh pre-cooled 70% ethanol, then dried, and finally dissolved in 50 μL of sterile water. Two microliters of the dissolved RNA was subjected to electrophoresis to check the quantity and quality. The extracted 2 μg RNA sample was reverse-transcribed into first-strand cDNA using reverse transcriptase (Takara Bio Inc.) according to the manufacturer’s instructions. The reaction conditions involved an oligo-(dT)_18_ primer (10 μM, 2μL), and the RNA sample was reverse transcribed at 42 °C for 60 min and then reverse transcribed at 70 °C for 15 min. Finally, 20 μL of first-strand cDNA was obtained. The purified cDNA of mature cuttlefish served as the template in further analyses.

### 3.3. Identification and Whole-Length Amplification of cDNA of SjFMRFamide 

According to the known sequences of *S. officinalis* (Y11246.1), degenerate primers FMRF-F/FMRF-R (see Table 1) were designed. The amplification reaction was carried out in a 25 μL volume including 10× PCR buffer (2.5 μL), 2.5 mM dNTPs (1 μL), 25 mM MgCl_2_ (1.5 μL), 10 μM FMRF-F (1 μL), and FMRF-R (1 μL) (see Table 1), 0.5 μg/μL template cDNA (1 μL), rTaq DNA polymerase (Takara Bio Inc.) (0.25 μL), and ddH_2_O (16.75 μL). The PCR amplification was conducted on a Thermal Cycler (Bio-Rad Laboratories, Inc., Hercules, CA, USA), and amplification conditions were as follows: 4 min at 94 °C, followed by 35 cycles of 30 s at 94 °C, 30 s at 50 °C, and 45 s at 72 °C with a final extension of 10 min at 72 °C. The 5′ and 3′ SjFMRFamide sequences were amplified via RACE. RACE library synthesis and PCRs were carried out by using the RACE kit (Ambion, Austin, TX, USA) and following the manufacturer’s instructions. RACE PCRs involved the following nested primers: FMRF-5′ outer and FMRF-5′ inner, FMRF-3′ outer, and FMRF-3′ inner (see Table 1). The resulting amplicons were ligated, cloned, and sequenced as outlined above.

### 3.4. Bioinformatics Analysis of SjFMRFamide

SjFMRFamide cDNA sequence was studied in several ways including the analysis of the protein-coding region, the analysis of potential signal sequences, theoretical MW prediction, *pI* calculation, the analysis of phosphorylation sites and glycosylation sites, transmembrane structure prediction, multiple sequence alignments, and phylogenic tree analysis. The software included Lasergene (DNASTAR, Inc., Madison, WI, USA), Scratch Protein Predictor and Predict Protein Web services, SignalP v4.0 (http://www.cbs.dtu.dk/services/SignalP-4.0/) [37], BLAST (https://blast.ncbi.nlm.nih.gov/Blast.cgi), ClustalW2 (http://www.ebi.ac.uk/Tools/msa/clustalw2/) [26], NetPhos 3.1 Server (http://www.cbs.dtu.dk/services/NetPhos/), NetNGlyc 1.0 Server (http:// www.cbs.dtu.dk/services/NetNGlyc/), TMHMM Server 2.0 (http://www.cbs.dtu.dk/services/TMHMM/), ExPASy ProtParam online tool (http://www.expasy.org/tools/protparam.html) [34], and MEGA 6.0 [38].

### 3.5. qRT-PCR of SjFMRFamide in Different Tissue

qRT-PCR was performed to research the expression levels of SjFMRFamide in tissues of the brain, gill, heart, muscle, liver, sexual gland (including testis & ovary), nidamental gland, and accessory nidamental gland of the matured cuttlefishes by using the SYBR Premix Ex Taq™ II Kit (Perfect Real Time; Takara Bio Inc.) as recommended by the manufacturer’s instructions for the 7500 Real-Time PCR System (Life Tech (applied biosystems)). A reaction mixture of 20 μL contained primers RT-FMRF-F and RT-FMRF-R (see Table 1) each at 0.8 μL (0.5 μM), 2× SYBR Premix Ex Taq™ II (Tli RNaseH Plus) (10 μL), 0.1 μg/μL cDNA sample (0.8 μL), ROX Reference Dye II (0.4 μL), and sterile Milli-Q water (7.2 μL). The amplification cycling conditions were conducted at 95 °C for 60 s, followed by 40 cycles of 10 s each at 95 °C, and completed for 45 s at 60 °C. The specificity of PCR was verified by melting curve analysis from 55 °C to 95 °C. The β-Actin gene (JN564496.1; primers RT-actin-F and RT-actin-R, Table 1) was chosen as the internal reference. Three biological replications were analyzed and each sample had three technical replications. 

The mRNA expression level detected in the heart tissue served as the standard while the threshold and Ct (threshold cycle) values acquired via RT PCR were used to analyze SjFMRFamide mRNA levels according to the 2^−ΔΔCt^ method. All the data were normalized and are presented as mean ± SE (*n* = 3). The data were processed in the SPSS software [31].

### 3.6. The In Situ Hybridization Assay of SjFMRFamide

Primers FMRF-probeF and FMRF-probeR (see Table 1) were designed according to the cDNA sequence of SjFMRFamide. The PCR was carried out with the brain cDNA as a template and the PCR product served as a template for in situ hybridization. The PCR setup was the same as that in Section 3.3. The following cycling conditions were employed: 94 °C for 5 min, 30 cycles of 94 °C for 30 s, 60 °C for 30 s, 72 °C for 30 s, and, lastly, 72 °C for 8 min. The purified template was used to prepare probes following the DIG RNA Labeling kit SP6/T7 kit (Roche Diagnostics, Mannheim, Germany) manufacturer’s instructions. The reaction was run in a 20 μL volume including 10× Transcription Buffer (2 μL), the 0.1μg/μL template (2 μL), 10× DIG Labeling mixture (2 μL), an RNA inhibitor (20 U/μL, 2 μL), T7 RNA polymerase (20 U/μL, 2 μL), and diethyl pyrocarbonate (DEPC)-treated water (10 μL), and incubated at 37 °C for 2 h. The reaction was stopped by the addition of 2 μL of 0.2 M EDTA. Afterward, 75 μL of precooled absolute ethanol was added to the centrifuge tube and the reaction mixture was mixed and kept at −20 °C for 3 h. After this, the solution was centrifuged at 12,000× *g* for 15 min at 4 °C and the supernatant was discarded. The resulting precipitate was washed with 100 μL of 70% ethanol and centrifuged at 7500× *g* for 5 min at 4 °C. After removal of the supernatant, the probe was finally dissolved in 50 μL of DEPC-treated water and 2 μL of the probe was subjected to electrophoresis. 

Brain tissues of cuttlefish (at the gonad development stage: phase V) were fixed with 4% paraformaldehyde (dissolved in 1× PBS) for 20 h and dehydrated in a graded series of ethanol solutions. Paraffin sections were dewaxed in dimethylbenzene for 5 min, rehydrated in PBS for 5 min, and treated with proteinase K at 37 °C for 20 min. The tissue slices were pre-hybridized in hybridization buffer for 1 h at 42 °C. Hybridization was performed at the same temperature with probe concentrations ranging between 0.5 and 5 μg/mL for 10–12 h. A digoxigenin (DIG)-labeled alkaline phosohatase (AP) antibody at a dilution of 1:500 in a blocking solution was incubated with the slices at 4 °C overnight. Color development in the NBT/BCIP/Alkaline Phosphatase buffer (Promega, Madison, WI, USA) solution took 3.5 h at room temperature. The tissue slices were washed with 1× PBS, sealed with glycerinum, and photographed by using the upright microscope (Olympus CX31, Olympus, Tokyo, Japan).

## 4. Conclusions

In conclusion, this is the first report of identification, characterization, and expression analysis of a FMRFamide-like peptide gene in the cuttlefish *S. japonica*. The analysis of phylogenetic and structural features will contribute to a better understanding evolutionary processes of mollusks. The similarity of mRNA expression of SjFMRFamide to that in other cephalopods and the in situ hybridization assay of the brain suggest that the protein may be related to many physiological regulatory mechanisms. The results may contribute to future studies of neuropeptide evolution or may prove useful for developing aquaculture methods for this species.

## Figures and Tables

**Table 1:** Primers used in SjFMRFamide cDNA identification and full-length amplification.

Primer Name	Sequence (5′–3′)	Position
FMRF-F	CAGCGTGWMCCTTYKTATCTT	172–192
FMRF-R	CAAAWAAGTCAATCWCGCAGG	1621–1641
5′-FMRF-outer	GATGACTTAGGTCCGACTAC	252–271
5′-FMRF-inner	GATGGAGCCGCACACACAGAC	193–213
3′-FMRF-outer	GCAAAGGGTGTCTGGAAGGT	1307–1326
3′-FMRF-inner	GACACACACGCAAACATACAC	1469–1489
RT-actin-F	TGAGAGGGAGATTGTGCGTG	815–834
RT-actin-R	GAACATAGATTCTGGAGCACGG	968–989
RT-FMRF-F	CGTCATCGCCATCTACTGTC	366–385
RT-FMRF-R	CGCTTGCTTCTCAGTCCATC	514–533
FMRF-probeF	CCCAAGCGTGATGCGTTGTTGGAGT	330–348
FMRF-probeR	CCGGAAGCGCTTGCTTCTCAGTCCATC	514–533

## Supplementary Materials

The following are available online.
