# Supplementary material for: Identification, Characterization, and Expression Analysis of a FMRFamide-Like Peptide Gene in the Common Chinese Cuttlefish (Sepiella japonica)
Source: Molecules. 2018 Mar 23;23(4):742. doi: 10.3390/molecules23040742 (PMC6017766; doi:10.3390/molecules23040742)
Supplement: Supplementary file 1 [file molecules-23-00742-s001.pdf]

Essential Items on the MIQE Checklist

Category	Item to be described/detailed	Location in Manuscript	Comments by Author
Sample	Type (blood, etc.)	Line 190	Tissue
	Method of dissection/procurement	Line 189	Anesthetized dissection
	Processing procedure	Line189-192	Quick dissection
	If frozen, how and how quickly?	Line 191	Liquid nitrogen, immediately
	If fixed, with what and how quickly?	------	Not fixed
	Storage conditions and duration	Line 192	−80 °C refrigerator, one month
Extraction	Method or instrument	Line 192-201	Traditional method
	Reagents/kits/modifications	Line 192	Reagents: TRIzol
	DNAse or RNAse treatment	------	Ribonuclease Inhibitor
	Evidence for lack of contamination (DNA or RNA)	Line 200-201	Electrophoresis and nucleic acid quantification
	Nucleic acid quantification	------	Purity (A260/A280): 1.9-2.1
	RNA integrity	------	Three complete bands of RNA
Reverse Transcription	Complete reaction conditions, including all components and their concentrations	Line 200-205	
	RNA amount and reaction volume	Line 200, 204	2 μg RNA, 20 μl reaction volume
	Priming oligo sequence(s)	Line 203	10 μM Oligo $(dT)_{18}$
	Cqs with and without reverse transcriptase	-----	Without reverse transcriptase
qPCR target	HUGO gene abbreviation	Line 61	SjFMRFamide
	Sequence accession number	Line 67	No. KJ933411
	Amplicon length	Table 1	366-533 (167bp)
	In silico specificity (BLAST)	------	Homology comparison with other species
	Location by exon/intron	------	Exon
	Identify the splice variants amplified	------	PCR
	All primer/probe sequences	Table 1	RT-FMRF-F and RT-FMRF-R
	Location and identity of any oligonucleotide modifications	------	No modifications
qPCR protocol	Complete reaction conditions, including all components and their concentrations	Line 238-243	
	cDNA/DNA amount and reaction volume	Line 240, 238	80 ng cDNA, 20 reaction volume
	Instrument identification and complete thermocycling parameters	Line 237-242	7500 Real-Time PCR System (Applied Biosystems, UK)
qPCR validation	Evidence for PCR specificity (gels, sequencing, or melting curves)	Line 242	Melting curves
	Template inhibition data (template titrations)	------	Recombinant Ribonuclease Inhibitor 10U
	For SYBR Green I reactions, the Cq of the no template control	------	Only test the Cq of the template

	Calibration curves with slope and intercept	------	Only saved the data
	PCR efficiency from the slope	------	Only saved the data
	r of the calibration curve	------	Did not save the calibration curve
	Evidence for the linear dynamic range	------	Did not save the linear dynamic range
	Evidence for the limit of detection	------	Did not save the limit of detection
	For multiplexed assays, the efficiency and limit of detection of each assay	------	Only one target gene was checked
Data analysis	qPCR analysis method/software	Line 248-249	$2^{-\Delta\Delta Ct}$ method, SPSS
	Method of Cq determination	Line 248	$2^{-\Delta\Delta Ct}$ method
	Results of no template controls	------	Only test the template
	Justification of number and choice of reference genes	Line 243	Based on others literature
	Normalization method	------	ΔCt=Ct(FMRF)-Ct(β-Actin)<br>ΔΔCt=ΔCt-ΔCt(heart)
	Number and stage (reverse transcription or qPCR) of technical replicates	Line 244-245	Three technical repeats
	Intra-assay variation in terms of concentration, not Cq	Line 240	cDNA concentration is 0.1 μg/μl
	Statistical methods/software	Line 148-149	LSD multiple comparisontest, SPSS
